# Association between macro- and microvascular damage and the triglyceride glucose index in community-dwelling elderly individuals: the Northern Shanghai Study

**DOI:** 10.1186/s12933-019-0898-x

**Published:** 2019-07-25

**Authors:** Song Zhao, Shikai Yu, Chen Chi, Ximin Fan, Jiamin Tang, Hongwei Ji, Jiadela Teliewubai, Yi Zhang, Yawei Xu

**Affiliations:** Department of Cardiology, Shanghai Tenth People’s Hospital, Tongji University School of Medicine, 301 Yanchang Road, Shanghai, 200072 China

**Keywords:** Triglyceride glucose index, Insulin resistance, Macrovascular damage, Microvascular damage

## Abstract

**Background:**

It has been reported that the triglyceride-glucose (TyG) index may serve as a simple and credible surrogate marker of insulin resistance (IR). However, its association with macrovascular and microvascular damage is unclear. Accordingly, the objective of the present study is to investigate the association of macrovascular and microvascular damage with the TyG index.

**Methods:**

A total of 2830 elderly participants from the Northern Shanghai Study (NSS) were enrolled. The TyG index was calculated as ln[fasting triglycerides (mg/dL) × fasting glucose (mg/dL)/2]. Parameters of vascular damage, including carotid-femoral pulse wave velocity (cf-PWV), brachial-ankle pulse wave velocity (ba-PWV), ankle–brachial index (ABI), carotid intima–media thickness (CMT), carotid plaque, estimated glomerular filtration rate (eGFR) and the urine albumin-to-creatinine ratio (UACR), were measured and calculated.

**Results:**

In univariate logistic regression, an increased TyG index was associated with a higher risk of cf-PWV > 10 m/s, ba-PWV > 1800 cm/s, ABI < 0.9, microalbuminuria (MAU) and chronic kidney disease (CKD). In multivariable logistic regression, there was a significant increase in the risk of cf-PWV > 10 m/s (OR = 1.86, 95% confidence interval [95% CI] 1.37–2.53, P_*for trend*_ < 0.001), ba-PWV > 1800 cm/s (OR = 1.39, [95% CI] 1.05–1.84, P_*for trend*_= 0.02), MAU (OR = 1.61, [95% CI] 1.22–2.13, P_*for trend*_ < 0.001) and CKD (OR = 1.67, [95% CI] 1.10–1.50, P_*for trend*_= 0.02) after adjustment for age, sex, BMI, waist circumference, smoking habit, hypertension, family history of premature CVD, diabetes, HDL-C, LDL-C, insulin therapy and statin therapy. However, no significant relationship was observed between the TyG index and lower extremity atherosclerosis, carotid hypertrophy or carotid plaque.

**Conclusion:**

An elevated TyG index was significantly associated with a higher risk of arterial stiffness and nephric microvascular damage. This conclusion lends support to the clinical significance of the TyG index for the assessment of vascular damage.

**Electronic supplementary material:**

The online version of this article (10.1186/s12933-019-0898-x) contains supplementary material, which is available to authorized users.

## Background

Cardiovascular disease (CVD) is the leading cause of mortality and morbidity worldwide [1]. Insulin resistance (IR) is suggested to be one of the most important risk factors for the development of CVD [2]. Many studies have shown that IR leads to vascular damage and CVD [2–5]. IR probably requires routine assessment in clinical practice. The homeostasis model assessment of insulin resistance (HOMA-IR) index is the most common method for evaluating insulin resistance in clinical practice [6]. However, this method of assessing insulin resistance is complex and expensive [7]. Therefore, many surrogate biomarkers have emerged [8]. Recently, a number of studies have shown that the triglyceride-glucose (TyG) index, calculated as ln[fasting triglycerides (mg/dL) × fasting glucose (mg/dL)/2], was significantly related to HOMA-IR [9, 10]. Thus, the TyG index could serve as a simple and credible surrogate marker of IR [9–12].

There are many clinical investigations regarding the significant association between vascular damage and the emerging TyG index. Irace et al. found that the TyG index was associated with carotid atherosclerosis [13]. Min et al. and ki-Bum et al. reported that the TyG index was independently associated with arterial stiffness in 3587 subjects and 2560 subjects, respectively, in Korean populations [14, 15]. A few prospective studies have shown that the TyG index was associated with new-onset diabetes [16], hypertension [17] and cardiovascular events [18]. It is well established that aging is significantly associated with the development of IR [19]. Therefore, our objective is to investigate the association between macrovascular (arterial stiffness, lower extremity atherosclerosis, carotid hypertrophy and carotid plaque) and microvascular damage (chronic kidney disease (CKD) and microalbuminuria (MAU)) and the emerging TyG index in an elderly community-dwelling Chinese population from the Northern Shanghai Study.

## Methods

### Participants

The Northern Shanghai Study (NSS) (ClinicalTrials.gov Identifier: NCT02368938) is a prospective, ongoing, and multistage population study. The study protocol has been published previously [20]. Briefly, participants were enrolled if they (1) were 65 years or older, (2) resided in the urban communities in Northern Shanghai, and (3) were willing to sign the informed consent and participate in long-term follow-up. The exclusion criteria were (1) New York Heart Association functional class IV or end-stage renal disease, (2) malignant tumor or life expectancy < 5 years, and (3) stroke history in the previous 3 months. From June 2014 to August 2018, 3092 subjects were invited, and 2830 (91.5%) participants were enrolled.

### Social, clinical, and biological parameters

A standard questionnaire was used to collect information, including age, sex, smoking habit, history of hypertension, previous cardiovascular diseases, history of diabetes mellitus, cerebrovascular disease, renal disease, and use of antihypertensive or antidiabetic drugs or insulin. Alcohol consumption habits were categorized into three groups: no alcohol consumption, moderate alcohol consumption (daily alcohol intake 0.10–19.99 g for women and 0.10–39.99 g for men), and excessive alcohol consumption (daily alcohol intake at least 20 g for women and at least 40 g for men) [21]. Body weight, body height and waist circumference (WC) were measured by professionals, and body mass index (BMI) was computed. Blood and urine samples were obtained when the participants were in a fasting state in the morning of the exam day. Biological markers were tested in the center of Shanghai Tenth People’s Hospital. The TyG index was calculated as ln[fasting triglycerides (mg/dL) × fasting glucose (mg/dL)/2]. eGFR was determined using the Chinese modified Chronic Kidney Disease Epidemiology Collaboration [22].

### Measurements and definitions of macro-or microvascular damage

The participants’ blood pressure was measured while they were in a supine position by a semiautomatic oscillometric device (Omron, Japan) after 5 min of rest. Blood pressure was calculated as the average value after repeating the measurement three times at intervals of 2 min. Hypertension was defined as systolic and/or diastolic blood pressure greater than 140/90 mmHg or the current use of an antihypertensive medication. Arterial stiffness was evaluated by carotid-femoral pulse wave velocity (cf-PWV) and brachial-ankle pulse wave velocity (ba-PWV). The carotid-femoral PWV (cf-PWV) was measured by applanation tonometry (SphygmoCor, AtCor Medical, Sydney, Australia). The brachial-ankle PWV (ba-PWV) and ankle–brachial index (ABI) were recorded automatically and calculated by a VP1000 system (Omron, Kyoto, Japan). Both cf-PWV and ba-PWV were tested after the participants rested for at least 5 min. Carotid intima–media thickness (CMT) and carotid plaque were determined by the MyLab 30 Gold cardiovascular system (ESAOTE SpA, Genoa, Italy) with a 7.5-MHz probe.

In the present study, macrovascular damage included arterial stiffness (cf-PWV > 10 m/s or ba-PWV > 1800 cm/s), lower extremity atherosclerosis (ABI < 0.9), carotid hypertrophy (CMT > 0.9 mm) and carotid plaque. Microvascular damage included CKD (eGFR ≤ 60 mL/min per 1.73 m^2^) and MAU (urinary albumin to creatinine ratio (UACR) > 30 mg/g).

### Statistical analysis

Quantitative parameters are shown as the mean ± standard deviation, and qualitative parameters are presented as numbers with the percentages in parentheses. The quantitative parameters of males and females were compared by Student’s t test, and qualitative parameters were compared by the Chi squared test. We used Pearson’s correlation to assess the correlation between the TyG index and cardiometabolic risk factors. The TyG index was grouped by quartiles when analyzing the relationship between the TyG index and macro- and microvascular damage risk through a logistic regression model. We performed two models: (a) crude; and (b) adjusted for age, sex, BMI, WC, smoking habit, hypertension, family history of premature CVD, diabetes, HDL-C, LDL-C, insulin therapy and statin therapy. We performed C-statistic values for the TyG index, fasting glucose, triglycerides, LDL-C, and non-HDL-C in a multivariable logistic regression model after adjusting for age, sex, BMI, WC, smoking habit, hypertension, family history of premature CVD, diabetes, HDL-C, insulin therapy and statin therapy. Statistical analyses were performed using SAS software version 9.4 (SAS Institute, Inc., Cary, North Carolina, USA). The significance level was set at P < 0.05.

## Results

### Participants

The characteristics of the participants are presented in Table 1. Of the participants, 1571 (55.51%) were men, 1862 (65.84%) had hypertension, and 650 (22.97%) had diabetes mellitus. A total of 432 men consumed alcohol (289 consumed a moderate amount of alcohol and 143 consumed an excessive amount of alcohol). Compared with men, women were significantly less likely to be smokers (2.16% vs. 54.73%, P < 0.001) and have a family history of premature CVD (24.22% vs. 19.06%, P = 0.001). Additionally, women had lower diastolic blood pressure (DBP) (77.4 ± 9.4 vs. 79.6 ± 9.6 mmHg, P < 0.001) and WC (85.40 ± 10.06 vs. 88.21 ± 9.43 cm, P < 0.001) and higher total cholesterol (195.8 ± 36.7 vs. 176.6 ± 36.9 mg/dL, P < 0.001), HDL-C (54.54 ± 13.41 vs. 47.58 ± 12.48 mg/dL, P < 0.001), LDL-C (119.8 ± 32.5 vs. 108.8 ± 31.4 mg/dL, P < 0.001), triglycerides (148.6 ± 95.2 vs. 137.7 ± 91.2 mg/dL, P = 0.002), and TyG index (8.78 ± 0.57 vs. 8.71 ± 0.57, P < 0.001). In terms of vascular damage, women showed lower CMT (0.62 ± 0.15 vs. 0.66 ± 0.17 mm, P < 0.001), lower incidence of carotid plaque (60.09% vs. 66.88%, P < 0.001) and lower extremity atherosclerosis (12.86% vs. 16.76%, P = 0.004) but higher ba-PWV (1907.4 ± 453.2 vs. 1827.5 ± 387.7 cm/s, P < 0.001), and eGFR (73.9 ± 12.4 vs. 67.8 ± 10.8 mL/min per 1.73 m^2^, P < 0.001).

**Table 1:** Characteristics of participants

	Overall (n = 2830)	Female (1571)	Male (1259)	P value
Age (years)	71.5 ± 6.2	71.7 ± 6.4	71.3 ± 6.1	0.17
Hypertension, n (%)	1862 (65.84)	1031 (65.63)	831 (66.00)	0.83
Diabetes mellitus, n (%)	650 (22.97)	361 (22.98)	289 (22.95)	0.99
Smoking habit, n (%)	722 (25.53)	34 (2.16)	688 (54.73)	< 0.001
Bachelor degree, n (%)	517 (18.27)	202 (12.86)	315 (25.02)	< 0.001
Daily drinkers, n (%)	432 (15.27)	0(0)	432 (34.31)	< 0.001
Moderate drinking, n	289	0	289	–
Excessive drinking, n	143	0	143	–
Family history of premature CVD, n (%)	617 (21.92)	378 (24.22)	239 (19.06)	0.001
SBP (mmHg)	135.1 ± 17.4	135.3 ± 18.2	134.8 ± 16.3	0.42
DBP (mmHg)	78.4 ± 9.6	77.4 ± 9.4	79.6 ± 9.6	< 0.001
Waist circumference (cm)	86.65 ± 9.88	85.40 ± 10.06	88.21 ± 9.43	< 0.001
Hip circumference (cm)	96.78 ± 7.30	96.69 ± 7.61	96.89 ± 6.90	0.46
BMI (kg/m^2^)	24.0 ± 3.6	24.0 ± 3.9	24.0 ± 3.3	0.59
Fasting glucose (mg/dL)	103.5 ± 32.0	102.9 ± 30.6	104.1 ± 33.8	0.36
Total cholesterol (mg/dL)	187.2 ± 38.0	195.8 ± 36.7	176.6 ± 36.9	< 0.001
HDL-C (mg/dL)	51.45 ± 13.46	54.54 ± 13.41	47.58 ± 12.48	< 0.001
LDL-C (mg/dL)	114.9 ± 32.5	119.8 ± 32.5	108.8 ± 31.4	<0.001
Triglyceride (mg/dL)	143.8 ± 93.6	148.6 ± 95.2	137.7 ± 91.2	0.002
Antihypertension agents, n (%)	1439 (50.85)	798 (50.80)	641 (50.91)	0.95
Hypoglycemic agents, n (%)	464 (16.40)	254 (16.17)	210 (16.68)	0.71
Insulin therapy, n (%)	99 (3.50)	55 (3.50)	44 (3.49)	0.99
Statin therapy, n (%)	485 (17.14)	287 (18.27)	198 (15.73)	0.07
Carotid plaque, n (%)	1785 (63.10)	944 (60.09)	842 (66.88)	< 0.001
ABI < 0.9, n (%)	413 (14.59)	202 (12.86)	211 (16.76)	0.004
CMT (mm)	0.64 ± 0.16	0.62 ± 0.15	0.66 ± 0.17	< 0.001
cf-PWV (m/s)	9.49 ± 2.31	9.53 ± 2.26	9.43 ± 2.37	0.24
ba-PWV (cm/s)	1871.9 ± 427.1	1907.4 ± 453.2	1827.5 ± 387.7	< 0.001
eGFR (mL/min per 1.73 m^2^)	71.2 ± 12.1	73.9 ± 12.4	67.8 ± 10.8	< 0.001
UACR (mg/g)	65.3 ± 153.5	69.1 ± 182.3	60.6 ± 107.6	0.13
TyG index	8.75 ± 0.57	8.78 ± 0.57	8.71 ± 0.57	< 0.001

Quantitative variables are shown as mean ± SD, and qualitative parameters are presented as numbers with the percentage in parentheses

*CVD* cardiovascular disease, *WC* waist circumference, *SBP* systolic blood pressure, *DBP* diastolic blood pressure, *BMI* body mass index, *HDL-C* high-density lipoprotein cholesterol, *LDL-C* low-density lipoprotein cholesterol, *ABI* ankle–brachial index, *CMT* carotid intima–media thickness, *cf-PWV* carotid to femoral aortic pulse wave velocity, *ba-PWV* brachial to ankle pulse wave velocity, *ABI* ankle brachial index, *eGFR* estimated glomerular filtration rate, *UACR* urine albumin to creatinine ratio, *TyG* triglyceride glucose

### Correlation between the TyG index and cardiometabolic risk factors

In the age-, sex- and education-adjusted correlation analysis, the TyG index was correlated with SBP (r = 0.13), DBP (r = 0.07), WC (r = 0.29), BMI (r = 0.25), HDL-C (r = − 0.49), LDL-C (r = 0.15) and total cholesterol (r = 0.124) (P < 0.001 for all) (Table 2).

**Table 2:** The age, sex and education-adjusted correlation between TyG index and cardiometabolic risk factors

	r	P value
SBP (mmHg)	0.13	< 0.001
DBP (mmHg)	0.07	< 0.001
WC (cm)	0.29	< 0.001
BMI (kg/m^2^)	0.25	< 0.001
HDL-C (mg/dL)	− 0.49	< 0.001
LDL-C (mg/dL)	0.15	< 0.001
TC (mg/dL)	0.24	< 0.001

*TyG* triglyceride glucose, *SBP* systolic blood pressure, *DBP* diastolic blood pressure, *WC* waist circumference, *BMI* body mass index, *HDL-C* high-density lipoprotein cholesterol, *LDL-C* low-density lipoprotein cholesterol, *TC* total cholesterol

### Association of macro- and microvascular damage with TyG index

**Fig. 1:**
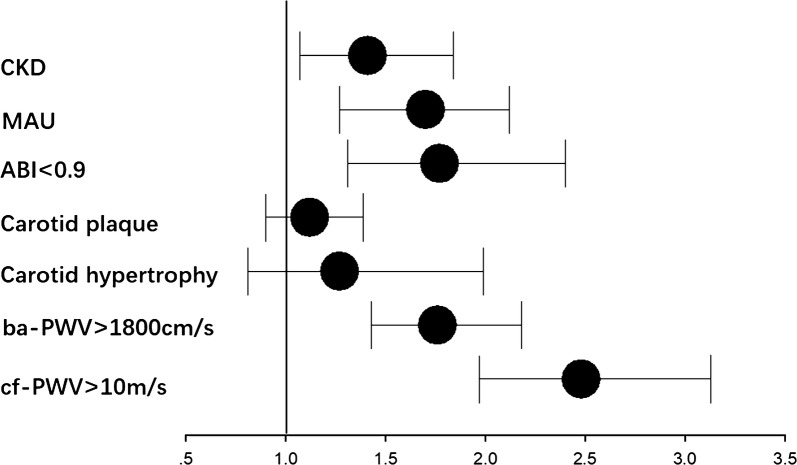
Q4 (fourth quartile) vs Ql (first quartile) odds ratio (OR) of TyG index for macro-and microvascular damage. TyG index levels for Q4 was associated with increased OR for cf-PWV>l0 m/s, ba- PWV > 1800 cm/s, ABl < 0.9, MAU and CKD, but not for carotid hypertrophy or carotid plaque in univariate logistic regression. *TyG* triglyceride glucose, *cf- PWV* carotid-femoral pulse wave velocity, *ba-PWV* brachial-ankle pulse wave velocity, *ABI* ankle–brachial index; *MAU* microalbuminuria, *CKD* chronic kidney disease

As shown in Table 3 and Fig. 1, in the univariate logistic regression, with Q1 (first quartile) set as the reference, the TyG index levels in Q4 (fourth quartile) were associated with an increased OR (odds ratio) for cf-PWV > 10 m/s (OR = 2.48, 95% confidence interval [95% CI] 1.97–3.13, P_*for trend*_ < 0.001), ba-PWV > 1800 cm/s (OR = 1.76, [95% CI] 1.43–2.18, P_*for trend*_ < 0.001), lower extremity atherosclerosis (OR = 1.77, [95% CI] 1.31–2.40, P_*for trend*_ < 0.001), MAU (OR = 1.70, [95% CI] 1.27–2.12, P_*for trend*_ < 0.001) and CKD (OR = 1.41, [95% CI] 1.07–1.84, P_*for trend*_ < 0.01) but not for carotid hypertrophy (OR = 1.27, [95% CI] 0.81–1.99, P_*for trend*_ = 0.26) or carotid plaque (OR = 1.12, [95% CI] 0.90–1.39, P_*for trend*_ = 0.17).

**Table 3:** Univariate logistic regression for risk of macro and microvascular damage according to the quartiles of the TyG index

	cf-PWV > 10 m/s	Ba-PWV > 1800 cm/s	Carotid hypertrophy	Carotid plaque	ABI < 0.9	MAU	CKD
Q1 (7.04 ≤ TyG index < 8.36)	1.00	1.00	1.00	1.00	1.00	1.00	1.00
Q2 (8.36 ≤ TyG index < 8.69)	1.57 (1.24–1.99)	1.40 (1.13–1.73)	1.08 (0.68–1.73)	1.10 (0.88–1.36)	1.21 (0.88–1.67)	1.06 (0.86–1.31)	1.02 (0.76–1.35)
Q3 (8.69 ≤ TyG index < 9.08)	1.76 (1.39–2.22)	1.52 (1.23–1.88)	1.21 (0.77–1.91)	1.26 (1.01–1.57)	1.36 (0.99–1.86)	1.13 (0.91–1.39)	1.12 (0.85–1.48)
Q4 (9.08 ≤ TyG index ≤ 11.63)	2.48 (1.97–3.13)	1.76 (1.43–2.18)	1.27 (0.81–1.99)	1.12 (0.90–1.39)	1.77 (1.31–2.40)	1.7 0(1.27–2.12)	1.41 (1.07–1.84)
P for tread	< 0.001	< 0.001	0.26	0.17	< 0.001	< 0.001	< 0.01

*TyG* triglyceride glucose, *cf-PWV* carotid-femoral pulse wave velocity, *ba-PWV* brachial-ankle pulse wave velocity, *ABI* ankle–brachial index, *MAU* microalbuminuria, *CKD* chronic kidney disease

**Fig. 2:**
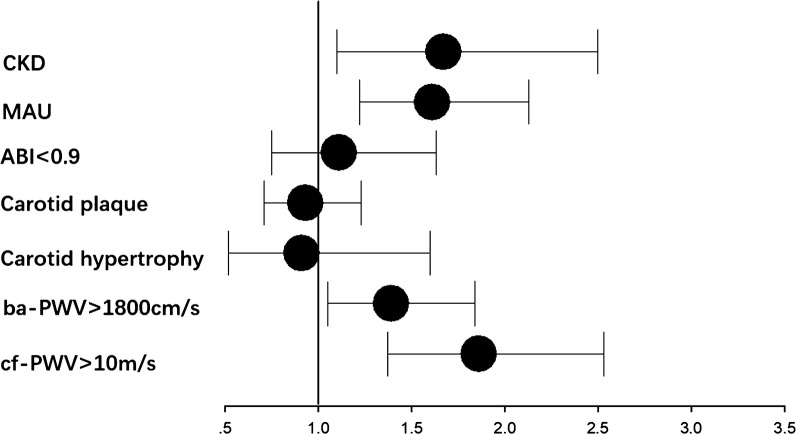
Q4 (fourth quartile) vs Ql (first quartile) odds ratio(OR) of TyG index for macro-and microvascular damage after adjustment for age, sex, BMI, WC, smoking habit, hypertension, family history of premature CVD, diabetes, HDL-C, LDL-C, insulin therapy and statin therapy. TyG index levels for Q4 was associated with increased OR for cf-PWV > 10 m/s, ba-PWV > 1800 cm/s, MAU and CKD, but not for ABl < 0.9, carotid hypertrophy or carotid plaque in multivariable logistic regression. *TyG* triglyceride glucose, *cf-PWV* carotid-femoral pulse wave velocity, *ba-PWV* brachial–ankle pulse wave velocity, *ABI* ankle–brachial index, *MAU* microalbuminuria, *CKD* chronic kidney disease, *BMI* body mass index, *WC* waist circumference, *CVD* cardiovascular disease, *HDL-C* high-density lipoprotein cholesterol, *LDL-C* low-density lipoprotein cholesterol

As shown in Table 4 and Fig. 2, after adjustment for age, sex, BMI, WC, smoking habit, hypertension, family history of premature CVD, diabetes, HDL-C, LDL-C, insulin therapy and statin therapy, we found that the TyG index levels in Q4 were associated with an increased OR for cf-PWV > 10 m/s (OR = 1.86, 95%, [95% CI] 1.37–2.53, P_*for trend*_ < 0.001), ba-PWV > 1800 cm/s (OR = 1.39, [95% CI] 1.05–1.84, P_*for trend*_ = 0.02), MAU (OR = 1.61, [95% CI] 1.22–2.13, P_*for trend*_ < 0.001) and CKD (OR = 1.67, [95% CI] 1.10–1.50, P_*for trend*_ = 0.02) compared with the TyG index levels in Q1, but the levels were not associated with lower extremity atherosclerosis (OR = 1.11, [95% CI] 0.75–1.63, P_*for trend*_ = 0.66), carotid hypertrophy (OR = 0.91, [95% CI] 0.52–1.60, P_*for trend*_ = 0.83) or carotid plaque (OR = 0.93, [95% CI] 0.71–1.23, P_*for trend*_ = 0.81).

**Table 4:** Multivariable logistic regression for risk of macro and microvascular vascular damage according to the quartiles of the TyG index

	cf-PWV > 10 m/s	ba-PWV > 1800 cm/s	Carotid hypertrophy	Carotid plaque	ABI < 0.9	MAU	CKD
Q1 (7.04 ≤ TyG index < 8.36)	1.00	1.00	1.00	1.00	1.00	1.00	1.00
Q2 (8.36 ≤ TyG index < 8.69)	1.51 (1.15–1.97)	1.30 (1.03–1.65)	0.89 (0.65–1.46)	1.10 (0.87–1.40)	1.05 (0.74–1.49)	0.98 (0.78–1.24)	1.19 (0.83–1.72)
Q3 (8.69 ≤ TyG index < 9.08)	1.66 (1.25–2.20)	1.48 (1.15–1.91)	0.94 (0.57–1.57)	1.22 (0.94–1.57)	1.01 (0.71–1.45)	1.13 (0.88–1.44)	1.24 (0.85–1.81)
Q4 (9.08 ≤ TyG index ≤ 11.63)	1.86 (1.37–2.53)	1.39 (1.05–1.84)	0.91 (0.52–1.60)	0.93 (0.71–1.23)	1.11 (0.75–1.63)	1.61 (1.22–2.13)	1.67 (1.10–2.50)
P for tread	< 0.001	0.02	0.83	0.81	0.66	< 0.001	0.02

Adjusted for age, sex, BMI, WC, smoking habit, hypertension, family history of premature CVD, diabetes, HDL-C, LDL-C, insulin and statin therapy

*TyG* triglyceride glucose, *cf-PWV* carotid-femoral pulse wave velocity, *ba-PWV* brachial-ankle pulse wave velocity, *ABI* ankle–brachial index, *MAU* microalbuminuria, *CKD* chronic kidney disease, *BMI* body mass index, *WC* waist circumference, *CVD* cardiovascular disease, *HDL-C* high-density lipoprotein cholesterol, *LDL-C* low-density lipoprotein cholesterol

Additionally, LDL-C and non-HDL-C were associated with carotid hypertrophy, carotid plaque and ABI < 0.9, while the TyG index, fasting glucose, and triglycerides were not. The TyG Index had a higher C-statistic for cf-PWV > 10 m/s and ba-PWV > 1800 cm/s but not for MAU or CKD after adjustment for age, sex, BMI, WC, smoking habit, hypertension, family history of premature CVD, diabetes, HDL-C, insulin therapy and statin therapy (Additional file 1).

In sensitivity analyses, the TyG index was significantly associated with cf-PWV > 10 m/s, ba-PWV > 1800 cm/s, and CKD in subjects without diabetes (n = 2180) and with cf-PWV > 10 m/s and CKD in subjects without hypertension (n = 968). However, the TyG index was less significantly associated with micro- and macrovascular damage in subjects without hypertension and diabetes (n = 803). This finding may be because of a much smaller sample size in these subgroups (Additional file 2).

## Discussion

The primary finding of the present study is that an increased TyG index was significantly associated with a higher risk of arterial stiffness (cf-PWV > 10 m/s or ba-PWV > 1800 cm/s), MAU and CKD but not lower extremity atherosclerosis, carotid hypertrophy or carotid plaque.

### TyG index and metabolic abnormalities

IR plays a role in metabolic abnormalities. The TyG index not only serves as a simple and reliable surrogate marker of IR but is also associated with metabolic abnormalities. Simental et al. found that the TyG index was correlated with IR assessed by HOMA-IR in healthy subjects [23]. Fernando et al. reported that the TyG index was an accessible and inexpensive way to identify insulin resistance in the general population [9]. Similar results were also found in obese adolescents [11, 12]. A higher TyG index was significantly associated with a higher risk of diabetes [16, 24, 25], hypertension [17, 26] and metabolic syndrome [27]. In the present study, we also found that the TyG index was significantly correlated with cardiometabolic risk factors.

### TyG index and macrovascular damage

IR is an important mechanism of type 2 diabetes (T2D). Macrovascular disease (CVD, stroke and peripheral arterial disease) is one of the complications of T2D. Macrovascular disease is the main cause of morbidity and mortality in patients with T2D [28]. Recently, it was reported that a history of peripheral arterial disease was associated with the risk of lower‑extremity amputation in patients with T2D [29]. It is very important to evaluate the structure and function of macrovasculature in the prevention and treatment of T2D. The TyG index may be associated with macrovascular damage. Irace et al. showed that the TyG index was significantly associated with CMT [13]. However, we did not find that the TyG index was associated with CMT or carotid plaque. It was reported that the TyG index was independently associated with arterial stiffness, as assessed by ba-PWV in a Korean population [14, 15]. In the present study, we found that the TyG index was associated with abnormalities of cf-PWV and ba-PWV. Cf-PWV is used as the gold standard of arterial stiffness. Our paper is the first to report the association between the TyG index and cf-PWV. It was reported that the prevalence of coronary artery calcification increased with an increasing TyG index [30, 31]. Moreover, subjects with a higher TyG index had a higher risk of developing cardiovascular events after adjusting for conventional CVD risk factors [18, 32].

### TyG index and microvascular damage

The UACR and eGFR are commonly used to assess renal function in patients with diabetes. MAU and CKD were associated with a higher risk of cardiovascular events [33]. Moreover, MAU is strongly associated with cardiac microvascular dysfunction [34]. However, the association between the TyG index and nephric microvascular damage remains unclear. Sikandar et al. reported that the TyG index demonstrated a higher positive linear correlation with UACR [35], but they did not perform further analysis. In the present study, we found that a higher TyG index was associated with a higher risk of CKD and MAU. The TyG index could be a predictor of incident CKD. The TyG index might play a role in nephric microvascular damage. In the future, more studies are needed to explore the relationship between the TyG index and microvascular damage, such as CKD, MAU, retinopathy, cardiac microvascular dysfunction and markers of endothelial injury.

### Limitations

The results of the present study need to be interpreted within the context of their limitations. First, this is a cross-sectional study, which limits what we can infer about the causality of the results. Second, the study included only older community-dwelling Chinese participants, so we should be cautious in extrapolating the present findings to other subjects. Third, we did not assess HOMA-IR in the present study, which is the gold standard method for measuring IR. However, the TyG index is certainly more convenient to measure in routine clinical practice. Finally, some metabolic conditions that might impact the results were not included in our investigation, such as primary triglyceride abnormalities.

## Conclusion

An elevated TyG index was significantly associated with a higher risk of arterial stiffness and nephric microvascular damage. This conclusion lends support to the clinical significance of the TyG index for the assessment of vascular damage.

## Additional files

**Additional file 1.** C-statistic for different lipid and glucose parameters in multivariable logistic regression model.

**Additional file 2.** TyG Index and macro and microvascular damage among patients without diabetes, without hypertension and without both diseases.

## Data Availability

The datasets used and/or analyzed in the study are available from the corresponding author upon reasonable request.

## Abbreviations

CVD: cardiovascular disease
IR: insulin resistance
HOMA-IR: homeostasis model assessment of insulin resistance
TyG: triglyceride glucose
MAU: microalbuminuria
CKD: chronic kidney disease
cf-PWV: carotid-femoral pulse wave velocity
ba-PWV: brachial-ankle pulse wave velocity
ABI: ankle–brachial index
CMT: carotid intima–media thickness
eGFR: estimated glomerular filtration rate
UACR: urine albumin to creatinine ratio
WC: waist circumference
BMI: body mass index
SBP: systolic blood pressure
HDL-C: high-density lipoprotein cholesterol
LDL-C: low-density lipoprotein cholesterol
OR: odds ratio
T2D: type 2 diabetes

## References

[1] Naghavi M, Abajobir AA, Abbafati C, Abbas KM, Abd-Allah F, Abera SF (2017). Global, regional, and national age-sex specific mortality for 264 causes of death, 1980–2016: a systematic analysis for the Global Burden of Disease Study 2016. Lancet.

[2] Bressler P, Bailey SR, Matsuda M, DeFronzo RA (1996). Insulin resistance and coronary artery disease. Diabetologia.

[3] Bonora E, Tessari R, Micciolo R, Zenere M, Targher G, Padovani R (1997). Intimal-medial thickness of the carotid artery in nondiabetic and NIDDM patients: relationship with insulin resistance. Diabetes Care.

[4] Bonora E, Formentini G, Calcaterra F, Lombardi S, Marini F, Zenari L (2002). HOMA-estimated insulin resistance is an independent predictor of cardiovascular disease in type 2 diabetic subjects: prospective data from the Verona Diabetes Complications Study. Diabetes Care.

[5] Hanley AJG, Williams K, Stern MP, Haffner SM (2002). Homeostasis model assessment of insulin resistance in relation to the incidence of cardiovascular disease—The San Antonio Heart Study. Diabetes Care.

[6] Matthews DR, Hosker JP, Rudenski AS, Naylor BA, Treacher DF, Turner RC (1985). Homeostasis model assessment—insulin resistance and beta-cell function from fasting plasma-glucose and insulin concentrations in man. Diabetologia.

[7] Bonora E, Targher G, Alberiche M, Bonadonna RC, Saggiani F, Zenere MB (2000). Homeostasis model assessment closely mirrors the glucose clamp technique in the assessment of insulin sensitivity: studies in subjects with various degrees of glucose tolerance and insulin sensitivity. Diabetes Care.

[8] Muniyappa R, Lee S, Chen H, Quon MJ (2008). Current approaches for assessing insulin sensitivity and resistance in vivo: advantages, limitations, and appropriate usage. Am J Physiol-Endocrinol Metab..

[9] Guerrero-Romero F, Simental-Mendia LE, Gonzalez-Ortiz M, Martinez-Abundis E, Ramos-Zavala MG, Hernandez-Gonzalez SO (2010). The product of triglycerides and glucose, a simple measure of insulin sensitivity. Comparison with the euglycemic-hyperinsulinemic clamp. J Clin Endocr Metab..

[10] Unger G, Benozzi SF, Perruzza F, Pennacchiotti GL (2014). Triglycerides and glucose index: a useful indicator of insulin resistance. Endocrinol Nutr..

[11] Nor NSM, Lee S, Bacha F, Tfayli H, Arslanian S (2016). Triglyceride glucose index as a surrogate measure of insulin sensitivity in obese adolescents with normoglycemia, prediabetes, and type 2 diabetes mellitus: comparison with the hyperinsulinemic-euglycemic clamp. Pediatr Diabetes..

[12] Kang B, Yang Y, Lee EY, Yang HK, Kim HS, Lim SY (2017). Triglycerides/glucose index is a useful surrogate marker of insulin resistance among adolescents. Int J Obesity..

[13] Irace C, Carallo C, Scavelli FB, De Franceschi MS, Esposito T, Tripolino C (2013). Markers of insulin resistance and carotid atherosclerosis. A comparison of the homeostasis model assessment and triglyceride glucose index. Int J Clin Pract..

[14] Lee SB, Ahn CW, Lee BK, Kang S, Nam JS, You JH (2018). Association between triglyceride glucose index and arterial stiffness in Korean adults. Cardiovasc Diabetol..

[15] Won K-B, Park G-M, Lee S-E, Cho I-J, Kim HC, Lee BK (2018). Relationship of insulin resistance estimated by triglyceride glucose index to arterial stiffness. Lipids Health Dis..

[16] Zhang M, Wang B, Liu Y, Sun X, Luo X, Wang C (2017). Cumulative increased risk of incident type 2 diabetes mellitus with increasing triglyceride glucose index in normal-weight people: The Rural Chinese Cohort Study. Cardiovasc Diabetol..

[17] Zheng R, Mao Y (2017). Triglyceride and glucose (TyG) index as a predictor of incident hypertension: a 9-year longitudinal population-based study. Lipids Health Dis..

[18] Sanchez-Inigo L, Navarro-Gonzalez D, Fernandez-Montero A, Pastrana-Delgado J, Alfredo Martinez J (2016). The TyG index may predict the development of cardiovascular events. Eur J Clin Invest.

[19] Liu J, Wu YY, Huang XM, Yang M, Zha BB, Wang F (2014). Ageing and type 2 diabetes in an elderly chinese population: the role of insulin resistance and beta cell dysfunction. Eur Rev Med Pharmacol..

[20] Ji H, Xiong J, Yu S, Chi C, Fan X, Bai B (2017). Northern Shanghai Study: cardiovascular risk and its associated factors in the Chinese elderly-a study protocol of a prospective study design. BMJ Open.

[21] Ezzati M, Rodgers A, Murray C. Comparative quantification of health risks. In: Global and regional burden of disease attributable to selected major risk factors. Geneva: World HealthOrganization;2004.http://apps.who.int/iris/bitstream/10665/42792/1/9241580348_eng_Volume1.pdf. Accessed 22 May 2019.

[22] Ji HW, Zhang H, Xiong J, Yu SK, Chi C, Bai B (2017). eGFRs from Asian-modified CKD-EPI and Chinese-modified CKD-EPI equations were associated better with hypertensive target organ damage in the community-dwelling elderly Chinese: the Northern Shanghai Study. Clin Interv Aging.

[23] Simental-Mendia LE, Rodriguez-Moran M, Guerrero-Romero F (2008). The product of fasting glucose and triglycerides as surrogate for identifying insulin resistance in apparently healthy subjects. Metab Syndr Relat Disord..

[24] Navarro-Gonzalez D, Sanchez-Inigo L, Pastrana-Delgado J, Fernandez-Montero A, Alfredo Martinez J (2016). Triglyceride-glucose index (TyG index) in comparison with fasting plasma glucose improved diabetes prediction in patients with normal fasting glucose: The Vascular-Metabolic CUN cohort. Prev Med.

[25] Low S, Khoo KCJ, Irwan B, Sum CF, Subramaniam T, Lim SC (2018). The role of triglyceride glucose index in development of Type 2 diabetes mellitus. Diabetes Res Clin Pract.

[26] Song J, Nie S-M, Chen X, Zhang J, Wu X-S (2017). Association and interaction between triglyceride-glucose index and obesity on risk of hypertension in middle-aged and elderly adults. Clin Exp Hypertens..

[27] Moon S, Park JS, Ahn Y (2017). The cut-off values of triglycerides and glucose index for metabolic syndrome in american and korean adolescents. J Korean Med Sci.

[28] Beckman JA, Paneni F, Cosentino F, Creager MA (2013). Diabetes and vascular disease: pathophysiology, clinical consequences, and medical therapy: part II. Eur Heart J..

[29] Schneider F, Saulnier PJ, Gand E, Desvergnes M, Lefort N, Thorin E (2018). Influence of micro- and macro-vascular disease and Tumor Necrosis Factor Receptor 1 on the level of lower-extremity amputation in patients with type 2 diabetes. Cardiovasc Diabetol..

[30] Kim MK, Ahn CW, Kang S, Nam JS, Kim KR, Park JS (2017). Relationship between the triglyceride glucose index and coronary artery calcification in Korean adults. Cardiovasc Diabetol..

[31] Lee EY, Yang HK, Lee J, Kang B, Yang Y, Lee SH (2016). Triglyceride glucose index, a marker of insulin resistance, is associated with coronary artery stenosis in asymptomatic subjects with type 2 diabetes. Lipids Health Dis..

[32] Jin JL, Cao YX, Wu LG, You XD, Guo YL, Wu NQ (2018). Triglyceride glucose index for predicting cardiovascular outcomes in patients with coronary artery disease. J Thorac Dis..

[33] Ninomiya T, Perkovic V, De Galan BE, Zoungas S, Pillai A, Jardine M (2009). Albuminuria and kidney function independently predict cardiovascular and renal outcomes in diabetes. J Am Soc Nephrol..

[34] Potier L, Chequer R, Roussel R, Mohammedi K, Sismail S, Hartemann A (2018). Relationship between cardiac microvascular dysfunction measured with 82Rubidium-PET and albuminuria in patients with diabetes mellitus. Cardiovasc Diabetol..

[35] Khan SH, Sobia F, Niazi NK, Manzoor SM, Fazal N, Ahmad F (2018). Metabolic clustering of risk factors: evaluation of Triglyceride-glucose index (TyG index) for evaluation of insulin resistance. Diabetol Metab Syndr..

