# Supplementary material for: Association between macro- and microvascular damage and the triglyceride glucose index in community-dwelling elderly individuals: the Northern Shanghai Study
Source: Cardiovasc Diabetol. 2019 Jul 25;18:95. doi: 10.1186/s12933-019-0898-x (PMC6657056; doi:10.1186/s12933-019-0898-x)
Supplement: Supplementary file 1 — Additional file 1. C-statistic for different lipid and glucose parameters in multivariable logistic regression model. [file 12933_2019_898_MOESM1_ESM.docx]

**Additional file 1: C-statistic for different lipid and glucose parameters in multivariable logistic regression model**

|  | **cf-PWV>10m/s** | | **ba-PWV>1800cm/s** | | **Carotid hypertrophy** | | **Carotid plaque** | | **ABI<0.9** | | **MAU** | | **CKD** | |
| --- | --- | --- | --- | --- | --- | --- | --- | --- | --- | --- | --- | --- | --- | --- |
|  | **P value** | **C-statistic** | **P value** | **C-statistic** | **P value** | **C-statistic** | **P value** | **C-statistic** | **P value** | **C-statistic** | **P value** | **C-statistic** | **P value** | **C-statistic** |
| **Fasting glucose** | **<0.001** | **0.731(0.711-0.751)** | **0.004** | **0.708(0.689-0.727)** | **0.85** | **0.659(0.615-0.704)** | **0.61** | **0.635(0.613-0.656)** | **0.80** | **0.688(0.659-0.716)** | **<0.001** | **0.647(0.626-0.666)** | **0.74** | **0.850(0.832-0.868)** |
| **Triglyceride** | **<0.001** | **0.731(0.712-0.751)** | **0.01** | **0.706(0.687-0.725)** | **0.99** | **0.659(0.615-0.704)** | **0.28** | **0.634(0.613-0.656)** | **0.90** | **0.689(0.660-0.717)** | **0.004** | **0.646(0.625-0.667)** | **<0.001** | **0.852(0.834-0.870)** |
| **LDL-C** | **<0.001** | **0.730(0.711-0.750)** | **0.19** | **0.705(0.686-0.724)** | **0.001** | **0.675(0.631-0.718)** | **0.001** | **0.641(0.619-0.662)** | **0.02** | **0.690(0.662-0.719)** | **0.46** | **0.643(0.622-0.664)** | **0.26** | **0.850(0.832-0.868)** |
| **non-HDL-C** | **<0.001** | **0.732(0.712-0.753)** | **0.005** | **0.707(0.687-0.726)** | **0.003** | **0.677(0.632-0.760)** | **0.04** | **0.638(0.616-0.659)** | **0.003** | **0.695(0.667-0.723)** | **0.002** | **0.649(0.628-0.670)** | **<0.001** | **0.852(0.834-0.870)** |
| **TyG index** | **<0.001** | **0.735(0.715-0.754)** | **<0.001** | **0.709(0.690-0.728)** | **0.81** | **0.659(0.614-0.704)** | **0.78** | **0.634(0.613-0.656)** | **0.49** | **0.688(0.659-0.716)** | **<0.001** | **0.646(0.625-0.667)** | **<0.001** | **0.852(0.834-0.870)** |

**Adjusted for age, sex, BMI, WC, smoking habit, hypertension, family history of premature CVD, diabetes, HDL-C, insulin and statin therapy. CVD: cardiovascular disease; LDL-C: low-density lipoprotein cholesterol; HDL-C: high-density lipoprotein cholesterol; TyG: triglyceride glucose; cf-PWV: carotid-femoral pulse wave velocity; ba-PWV: brachial-ankle pulse wave velocity; ABI: ankle–brachial index; MAU: microalbuminuria; CKD: chronic kidney disease; WC: waist circumference; BMI: body mass index.**
