# Supplementary material for: Association between macro- and microvascular damage and the triglyceride glucose index in community-dwelling elderly individuals: the Northern Shanghai Study
Source: Cardiovasc Diabetol. 2019 Jul 25;18:95. doi: 10.1186/s12933-019-0898-x (PMC6657056; doi:10.1186/s12933-019-0898-x)
Supplement: Supplementary file 2 — Additional file 2. TyG Index and macro and microvascular damage among patients without diabetes, without hypertension and without both diseases. [file 12933_2019_898_MOESM2_ESM.docx]

**Additional file 2: TyG Index and macro and microvascular damage among patients without diabetes, without hypertension and without both diseases**

|  | **without diabetes* (2180)** | | **without hypertension **(968)** | | **without both diseases*** (803)** | |
| --- | --- | --- | --- | --- | --- | --- |
|  | **TyG Index** | | | | | |
|  | **β±SE** | **P value** | **β±SE** | **P value** | **β±SE** | **P value** |
| **cf-PWV>10m/s** | **0.40±0.13** | **0.002** | **0.49±0.20** | **0.01** | **0.49±0.26** | **0.07** |
| **ba-PWV>1800cm/s** | **0.29±0.12** | **0.01** | **0.21±0.17** | **0.21** | **0.11±0.22** | **0.63** |
| **Carotid hypertrophy** | **-0.31±0.24** | **0.20** | **0.18±0.36** | **0.62** | **-0.26±0.47** | **0.58** |
| **Carotid plaque** | **-0.05±0.11** | **0.67** | **-0.20±0.16** | **0.19** | **-0.05±0.19** | **0.80** |
| **ABI<0.9** | **-0.01±0.16** | **0.95** | **-0.08±0.23** | **0.73** | **-0.06±0.29** | **0.84** |
| **MAU** | **0.20±0.11** | **0.07** | **0.16±0.15** | **0.31** | **0.11±0,19** | **0.57** |
| **CKD** | **0.43±0.17** | **0.01** | **0.65±0.28** | **0.02** | **0.54±0.35** | **0.12** |

***Adjusted for age, sex, BMI, WC, smoking habit, family history of premature CVD, HDL-C, LDL-C, statin therapy, hypertension.**

****Adjusted for age, sex, BMI, WC, smoking habit, family history of premature CVD, HDL-C, LDL-C, statin therapy, diabetes, insulin therapy.**

***** Adjusted for age, sex, BMI, WC, smoking habit, family history of premature CVD, HDL-C, LDL-C, statin therapy.**

**TyG: triglyceride glucose; cf-PWV: carotid-femoral pulse wave velocity; ba-PWV: brachial-ankle pulse wave velocity; ABI: ankle–brachial index; MAU: microalbuminuria; CKD: chronic kidney disease; BMI: body mass index ; WC: waist circumference; CVD: cardiovascular disease; HDL-C: high-density lipoprotein cholesterol; LDL-C: low-density lipoprotein cholesterol.**
